# Association between OH/ECW and echocardiographic parameters in CKD5 patients not undergoing dialysis

**DOI:** 10.1371/journal.pone.0195202

**Published:** 2018-04-09

**Authors:** Byoung-Geun Han, Shin Han Song, Jin Sae Yoo, Hyeoncheol Park, Juwon Kim, Eunhee Choi

**Affiliations:** 1 Department of Nephrology, Yonsei University Wonju College of Medicine, Wonju, Kang-won, S. Korea; 2 Department of Laboratory Medicine, Yonsei University Wonju College of Medicine, Wonju, Kang-won, S. Korea; 3 Institute of Lifestyle Medicine, Yonsei University Wonju College of Medicine, Wonju, Kang-won, S. Korea; Istituto Di Ricerche Farmacologiche Mario Negri, ITALY

## Abstract

**Background:**

Echocardiography is the most valuable tool for assessing cardiac abnormalities of chronic kidney disease (CKD) patients even though it has its limitations, including high equipment cost and the need for specialized personnel. Assessment of volume status is important not only for volume management, but also for prevention of cardiovascular disease of the CKD patients. Recently, bioimpedance is gaining acceptance as a way to quantitatively assess patient hydration status at bedside.

**Methods:**

127 patients who were admitted for planning their first dialysis treatment were enrolled. The echocardiography and bioimpedance spectroscopy (BIS) were performed. The association between echocardiographic data and clinical values such as NT-proBNP and OH/ECW was examined.

**Results:**

OH/ECW, which indicates relative fluid overload, was positively associated with LA dimension (r = 0.25, P = 0.007), LAVI (r = 0.32, P < 0.001), and E/e´ ratio (r = 0.38, P < 0.001). While OH/ECW was not significantly associated with echocardiographic values such as LVEDD, LVEDV, LVMI, and LVEF, NT-proBNP were significantly associated with all echocardiographic parameters. Multivariate logistic regression analysis showed E/e´ ratio (odds ratio, 1.14 [95% confidence interval (CI), 1.01 to 1.29]; P = 0.031), NT-proBNP (odds ratio, 4.78 [95% CI, 1.51 to 15.11]; P = 0.008), and albumin (odds ratio, 0.22 [95% CI, 0.08 to 0.66]; P = 0.007) were significantly associated with OH/ECW.

**Conclusions:**

Since OH/ECW measured by BIS is associated with echocardiographic parameters related to diastolic dysfunction, preliminary screening through laboratory findings, including serum albumin in conjunction with OH/ECW and NT-proBNP, may find patient with risk of diastolic dysfunction. Our study suggests that a timely detection of fluid overload in patients with CKD as well as their proper treatment may help reduce diastolic dysfunction. Further research may be needed to validate the consistency of this association across other stages of CKD.

## Introduction

Early detection of cardiac abnormalities in patient with chronic kidney diseases (CKD) can prompt early intervention and may result in improvements in cardiac prognosis. There are various tools for detecting cardiovascular disease, from simple blood pressure or arterial stiffness measurement to more advanced screening via computed tomography, magnetic resonance imaging, and echocardiography. NT-proBNP, which has been used as a biomarker for heart failure in the general population, was found to be elevated in CKD patients due to decreased renal excretion [1]. The use of NT-proBNP alone in screening for cardiac structural and functional abnormalities in CKD patients is not currently recommended, because there is no cut-off value recommended for different stage of CKD. Echocardiography is the most appropriate monitoring tool for patients with CKD [2]. The Kidney Disease Outcomes Quality Initiative (KDOQI) guidelines recommend echocardiograms for all patients at the initiation of dialysis (ideally within 1–3 months of dialysis initiation) and at 3-yearly intervals thereafter [3]. Nonetheless, there are no such guidelines for dialysis naive patients with renal insufficiency.

Assessment of volume status in patients with renal insufficiency, including those undergoing dialysis, is important not only for short-term volume management but also for long-term prevention of cardiovascular disease. However, the traditional methods have several limitations for detecting the degree and severity of overhydration [4]. Bioimpedance spectroscopy (BIS) can be used to monitor extracellular and intracellular water (ECW and ICW) status, and several recent studies have examined the clinical significance of overhydration value (OH) on BIS and its derivatives, such OH/ECW [5–7]. Maintaining a fluid balance is one of the crucial factor in reducing the risk of cardiovascular events associated with volume overload. Detection of volume overload prior to its clinical manifestation including edema is imperative, and a non-invasive, rapid and relatively affordable method to quantitatively assess the volume status of the patient is necessary.

Herein, we investigated the relationship between the echocardiography and the parameters of BIS in dialysis naive CKD5 patients.

## Materials and methods

### 1. Patients

This is a retrospective cohort analysis where BIS, echocardiography, and serum measurement of NT-proBNP were performed on the patient who were admitted for planning their dialysis treatment. All tests were performed at the same time before the first dialysis, either hemodialysis or peritoneal dialysis. Between October 2014 to April 2017, 141 consecutive patients were included in the current study. Patients with malignancy (n = 5), infection (n = 2), liver cirrhosis (n = 1), atrial fibrillation (n = 1), valvular heart disease (n = 1), abnormal left ventricular (LV) segmental wall motion (n = 2) or acute kidney injury (n = 2) were excluded from analysis. Among 127 patients who were finally enrolled, three patients whose LV ejection fraction (LVEF) was less than 45% were included. The study was approved by the Institutional Review Board of Yonsei University Wonju Severance Christian Hospital based on the Helsinki declaration. All participants completed the consent form.

### 2. Echocardiographic assessment

Echocardiography was performed in harmonic imaging mode using a 3-MHz transducer and commercial ultrasound system (Vivid-7; General Electric-Vingmed, Milwaukee, WI, USA).

LV internal dimensions, LV wall thickness, LV fractional shortening (LVFS) and LVEF were measured according to the recommendations of the American Society of Echocardiography [8]. LV mass was calculated using the following formula:

$$\text{LV}\;\text{mass}\;\left(\text{g}\right)=1.04\times \left({\left[\text{PWTd}+\text{SWTd}+\text{LVDd}\right]}^{3}\times {\left[\text{LVEDD}\right]}^{3}\right)\times 0.8+0.6$$

where PWTd and SWTd are the posterior and septal wall thickness at end-diastole, respectively, and LVEDD is the M-mode LV dimension with the short axis view at end-diastole. To correct for body surface area, the LV mass index (LVMI) was calculated as LV mass/body surface area [9]. Body surface area (BSA) was calculated using the formula:

$$\text{BSA}\;=\;0.007184\times {\text{weight}}^{0.425}\times {\text{height}}^{0.725}\left({\text{m}}^{2}\right).$$

The left atrial (LA) dimension was measured using 2D-guided M-mode echocardiography via the parasternal short-axis view at the base of the heart. Three LA dimensions were used to calculate the LA volume as an ellipse using the following formula:

LAvolume=(π/6)(SA1×SA2×LA)

where SA1 is the M-mode LA dimension, and SA2 and LA are measurements of the short- and long-axis with the apical four-chamber view at ventricular end-systole, respectively [8]. The LA volume index (LAVI) was calculated by dividing LA volume/BSA.

Transmitral inflow velocities were measured using pulsed-wave Doppler in the apical four-chamber view with the sample volume placed at the mitral valve leaflet tips. Transmitral early diastolic (E wave) velocities were measured. Tissue Doppler imaging (TDI) in the apical four-chamber view was used to measure LV myocardial velocities, with the sample volume placed at the septal mitral annulus. We measured the peak early (e´) diastolic mitral annular velocity and calculated E/e´ ratio [10, 11].

Echocardiography was performed by trained specialists who were completely blinded to patient information.

### 3. Evaluation of fluid status

The BIS using the Body Composition Monitor^™^ (BCM^™^, Fresenius Medical Care, Bad Homburg, Germany) was performed. BCM^™^ utilizes alternating electric currents across 50 different frequencies from 5 to 1000 kHz for the measurement of fluid status. ECW, ICW and total body water (TBW) were automatically calculated. The OH can also be calculated from the difference between the normal expected ECW and actually measured ECW. Whereas BIS can measure OH, bioimpedance analysis (BIA) using a single or several (5 to 10) electric current frequencies cannot be used to calculate OH. The validity of BIS in a study of healthy populations and dialysis patients has already been demonstrated in comparison to standard measurement methods [12]. OH/ECW greater than or equal to 15% was defined as fluid overload [13, 14].

### 4. Laboratory evaluations

The laboratory study was performed before the first dialysis application. NT-proBNP was measured using electrochemiluminescence immunoassay (ECLIA) on Modular Analytics E170 (Roche Diagnostics, Mannheim, Germany).

### 5. Statistical analyses

Statistical analysis was performed using SAS version 9.4 (SAS Inc., Cary, NC, USA). Descriptive statistics were described as mean ± standard deviation for continuous variables. NT-proBNP was logarithmically transformed as it was not normally distributed. OH/ECW was categorized into two groups: OH/ECW <15%, and OH/ECW ≥15% (fluid overload). Differences in clinical variables between the two groups were tested with two-sample *t*-test for continuous variables. The nominal variables (sex, presence of diabetes, use of hypertension medication, and use of medication for diuresis) were compared using Chi-square test or Fisher’s exact test. Pearson’s correlation analysis was used to examine correlations between volume markers (OH, OH/ECW, NT-proBNP) and echocardiographic variables. We also examined the associations of LAVI, E/e´ ratio, NT-proBNP, and albumin with OH/ECW using multivariate logistic regression models. Statistical analyses were performed after dichotomizing the patients according to the median NT-proBNP value. We tested the predictive accuracy of a logistic regression model using the c-statistic (equivalent to the area under the Receiver Operating Characteristic curve). Goodness of fit for model was assessed using the Hosmer-Lemeshow test, whereby we considered a value of P < 0.05 to indicate that the model had a poor fit. In this analysis, we adjusted for age and sex (model 1) and for diuretics use and estimated glomerular filtration rate (eGFR) (model 2). Statistical significance was defined as P < 0.05.

## Results

### 1. Patients’ characteristics

The characteristics of the study population are shown in Table 1. Patients with OH/ECW ≥15%, were receiving more diuretics and had more often diabetes. The mean age of male and female patients was 61.63 ± 12.50 years and 60.19 ± 11.55 years, respectively, and this difference was not statistically significant. The median [interquartile range (IQR)] OH and OH/ECW were 2.5 (0.7–5.2) L and 14.45 (0.05–27.95) %, respectively. The median NT-proBNP were 4,003.5 (816.5–12,428.75) pg/ml.

**Table 1:** Demographic findings of study patients.

Variables		Total (N = 127)	OH/ECW <15%	OH/ECW ≥15%	P-value
			N = 66	N = 61	
Age, years		61.04±12.10	61.53±11.59	60.51±12.70	0.64
Sex	Men	75 (59.1%)	39 (52.0%)	36 (48.0%)	0.99
	Women	52 (40.9%)	27 (51.9%)	25 (48.1%)	
Diabetes	Yes	72 (56.7%)	28 (38.9%)	44 (61.1%)	0.001
	No	55 (43.3%)	38 (69.1%)	17 (30.9%)	
BP medication	Yes	119 (93.7%)	61 (51.3%)	58 (48.7%)	0.72
	No	8 (6.3%)	5 (62.5%)	3 (37.5%)	
Diuretics	Yes	86 (72.3%)	38 (44.2%)	48 (55.8%)	0.013
	No	33 (27.7%)	23 (69.7%)	10 (30.3%)	
SBP, mmHg		142.36±18.97	140.47±19.16	144.34±18.73	0.26
DBP, mmHg		78.28±12.09	77.88±12.86	78.69±11.32	0.56
eGFR, mL/min/1.73 m^2^		5.96±2.55	6.40±2.43	5.49±2.60	0.04

### 2. Differences according to OH/ECW

**Fig 1:**
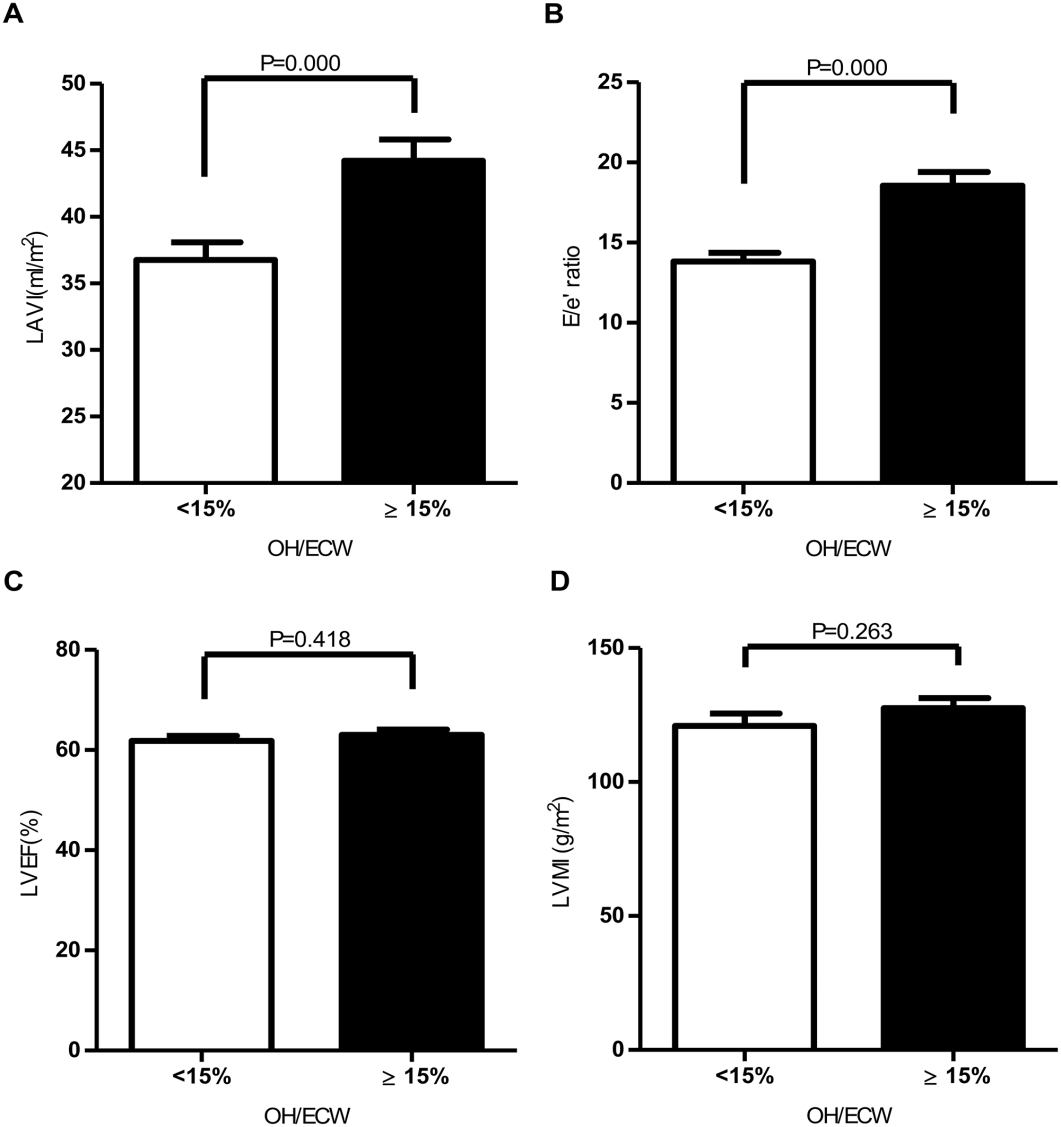
Comparison of left atrial volume index (LAVI) (A), E/e´ ratio (B), left ventricular ejection fraction (LVEF) (C), and left ventricular mass index (LVMI) (D) between non-overhydrated and overhydrated groups.

Among the echocardiographic parameters, LAVI and E/e´ ratio were significantly greater in fluid overloaded group, while LA dimension, LVEDD, LVEDV, LVMI, and LVEF were not significant (Fig 1). Serum parameters that were significantly different between the two groups are shown in Table 2. NT-proBNP was also significantly higher in fluid overloaded group compared to the non-overhydrated group (median = 7,976 pg/ml, IQR = 16,788 pg/ml versus median = 1,251.8 pg/ml, IQR = 3,045.2 pg/ml).

**Table 2:** Comparison of CKD patients with and without volume overload according to binary OH/ECW.

variables	Total (N = 127)	OH/ECW		P-value
		<15% (N = 66)	≥15% (N = 61)	
LA dimension (cm)	4.76±0.45	4.69±0.43	4.84±0.46	0.07
LAVI (ml/m^2^)	40.40±11.94	36.77±10.29	44.20±12.44	0.001
E/e´ ratio	16.13±5.96	13.82±4.23	18.56±6.56	<0.001
LVEDD (cm)	5.50±0.57	5.46±0.55	5.55±0.59	0.42
LVEDV (ml)	151.05±33.86	147.61±34.23	154.66±33.37	0.25
LVMI (g/m^2^)	124.17±32.86	120.90±36.20	127.61±28.85	0.26
LVFS (%)	34.51±5.71	34.16±5.59	34.88±5.85	0.49
LVEF (%)	62.40±8.18	61.81±7.93	63.02±8.47	0.42
NT-proBNP (pg/mL) *	4,003.5 (816.5–12,428.75)	1,251.8 (417.8–3463)	7,976 (4,166–20,954)	<0.001
Hemoglobin (g/dL)	9.07±1.37	9.41±9,316	13,284±12,102	0.004
CRP (mg/L)	1.75±3.17	1.44±2.74	2.09±3.59	0.27
Protein (g/dL)	6.12±0.74	6.37±0.62	5.83±0.76	<0.001
Albumin (g/dL)	3.41±0.56	3.65±0.46	3.15±0.53	<0.001
Calcium (mg/dL)	7.46±1.05	7.66±1.17	7.24±0.86	0.026
Phosphorus (mg/dL)	6.03±1.61	5.72±1.41	6.37±1.74	0.023

* Median (Interquartile range)

### 3. Correlation between laboratory, echocardiographic parameters and markers of volume status

NT-proBNP, which is known to reflect volume status and LV functional status, was positively correlated with OH and OH/ECW, while protein, albumin, calcium showed an inverse correlation (S1 Table).

OH, which indicates absolute volume overload, was significantly correlated with LA dimension, LAVI, E/e´ ratio, and LVEDD while OH/ECW, which indicates relative fluid overload, was positively associated with LA dimension, LAVI, and E/e´ ratio. While OH/ECW was not significantly associated with echocardiographic values related to systolic function (LVEF), NT-proBNP was significantly associated with all echocardiographic parameters (Table 3).

**Table 3:** Correlation of OH, OH/ECW, and NT-pro BNP with echocardiographic parameters.

variables	OH		OH/ECW		NT-proBNP	
	Corr. coeff.	P-value	Corr. coeff.	P-value	Corr. coeff.	P-value
LA dimension (cm)	0.22	0.017	0.25	0.007	0.25	0.007
LAVI (ml/m^2^)	0.21	0.020	0.32	<0.001	0.57	<0.001
E/e´ ratio	0.28	0.002	0.38	<0.001	0.46	<0.001
LVEDD (cm)	0.19	0.039	0.18	0.05	0.36	<0.001
LVEDV (ml)	0.18	0.046	0.18	0.05	0.41	<0.001
LVMI (g/m^2^)	0.12	0.20	0.15	0.11	0.38	<0.001
LVFS (%)	0.05	0.61	0.03	0.77	-0.46	<0.001
LVEF (%)	0.06	0.55	0.04	0.70	-0.48	<0.001

Corr. coeff., correlation coefficient

### 4. Multivariate analysis using logistic regression

Among the significantly different variables between the two OH/ECW groups, LAVI, E/e´ ratio, and NT-proBNP were positively associated with fluid overload, while the negative association was found for albumin in unadjusted model. The statistical analyses were performed after dichotomizing the patients according to the median NT-proBNP value of 4,003.5 (IQR, 816.5–12,428.75) pg/ml (Table 4). E/e´ ratio, NT-proBNP, and albumin showed significant odds ratio in model 1 and model 2. The Hosmer–Lemeshow tests showed significant goodness of fit for model 1 (P = 0.257) and model 2 (P = 0.775). Model 2 showed high capacity for predicting overhydration. The c-statistic was 0.88 (95% CI 0.814 ~0.942, P = 0.000) and 0.89 (95% CI 0.825 ~0.946, P = 0.000) for model 1 and model 2, respectively.

**Table 4:** Multivariate analysis using logistic regression: Predictive factors of being in the OH/ECW>15% group.

variables	Univariate analysis		Model 1		Model 2	
	OR (95%CI)	P-value	OR (95%CI)	P-value	OR (95%CI)	P-value
LAVI (ml/m^2^)	1.06 (1.02~1.09)	0.001	1.00 (0.95~1.05)	0.979	0.99 (0.94~1.04)	0.699
E/e´ ratio	1.20 (1.10~1.31)	<0.001	1.15 (1.03~1.30)	0.018	1.14 (1.01~1.29)	0.031
NT-proBNP <4,004 (pg/mL)	Reference		Reference		Reference	
NT-proBNP ≥4,004 (pg/mL)	11.81 (5.08~27.46)	<0.001	5.13 (1.69~15.56)	0.004	4.78 (1.51~15.11)	0.008
Albumin (g/dL)	0.12 (0.05~0.30)	<0.001	0.22 (0.08~0.66)	0.007	0.22 (0.08~0.66)	0.007

Model 1: Adjusted for age and sex.

Model 2: Adjusted for age, sex, diuretics use, and eGFR.

## Discussion

Among the data provided by BIS, OH (in liters), OH/ECW (in %), and ECW/TBW can be used for the assessment of volume status. An OH/ECW cutoff of 15% is correlated with an OH value of approximately 2.4 liters, which is generally accepted as the threshold for fluid overload, and in one study, it was suggested as a predictor of mortality in dialysis patients [14, 15]. Although the cut-off value of the OH/ECW for fluid overload differs between studies, most of the studies were conducted on the basis of 15% [13, 16, 17].

Various studies of NT-proBNP were reported in the field of nephrology and the elevated NT-proBNP was associated with an increased incidence of heart failure [18]. Booth *et al*. reported NT-proBNP to be associated with volume overload, but not with cardiac dysfunction [19]. Logarithmically-transformed NT-proBNP was significantly greater in CKD patients in fluid overloaded group (OH/ECW ≥15%) [20]. However, it remains unclear whether NT-proBNP itself reflects volume status or whether it is a by-product of structural damage to the heart due to fluid overload [1]. Dialysis therapy may also affect the serum level of NT-proBNP. Only three patients had systolic heart failure with an LVEF of less than 45% in this study. Statistically, there was no difference in LVEF between the two groups. Our results suggest that NT-proBNP is a marker that reflects fluid imbalance rather than heart failure in this study. In a study predicting cardiovascular outcomes, NT-proBNP was shown to outperform echocardiography, but data on the association with the volume status was not provided [21].

Most prior studies on the association between volume status and echocardiography have included patients who are already undergoing dialysis and thus prone to constant change of hemodynamic and volume status due to pre-formed arteriovenous fistula, hemodialysis, or constant exchange of peritoneal dialysis fluid. In this study, we only included those who have not undergone dialysis [22, 23]. In general, LAVI, rather than LA dimension, is a better predictor of cardiovascular events, such as atrial fibrillation, heart failure, myocardial infarction [24]. In patients undergoing dialysis treatment, LAVI was the best predictor of mortality and was significantly associated with fluid overload [25, 26]. Enrolled patients in this study were all dialysis naive (CKD) patients.

In this study, we found that NT-proBNP is associated with all echocardiographic parameters, while OH/ECW is associated only with LA dimension, LAVI, and E/e´ ratio. These three factors also differed between the two groups, but the variable reflecting systolic function (LVEF) did not show significant difference. Of note, mean LA dimension and mean LAVI were both greater than the reference values (4.0 cm for LA dimension and 34 mL/m^2^ for LAVI) even in CKD patients with OH/ECW less than 15%, implying that cardiac structural and functional abnormalities may manifest in the early stages of CKD regardless of the volume status.

E/e´ ratio is a marker of diastolic dysfunction, and a cut-off value of 15 is an important diagnostic criterion of heart failure with preserved ejection fraction [27]. In our study, the E/e´ ratio was significantly higher in CKD patients with OH/ECW ≥15% (18.56±6.56 vs. 13.82±4.23, p < 0.05), suggesting the possibility that the diastolic heart failure is higher in fluid overloaded patients compare to the non-overhydrated patients. While LAVI was associated with OH/ECW and was significantly different between the two groups, the association was not maintained in the adjusted model. However, the statistical significance of E/e´ ratio was maintained throughout the logistic analysis (P = 0.018 in model 1, P = 0.031 in model 2). This suggests that E/e´ ratio is more appropriate for assessing diastolic function than LAVI, and a stronger clinical significance may be imbued when it is used in conjunction with NT-proBNP (P = 0.008 in model 2).

Our study has several limitations. First, it included a relatively small number of patients from a single tertiary referral hospital. Second, a full workup on coronary artery disease was not performed. Also, the laboratory data before and after the intervention could not be compared because not all patients underwent the exactly same tests for the pre-dialysis screening test. Lastly, the effects of confounding factors could not be completely excluded although the multivariate models were used. Despite these limitations, this study included relatively homogeneous population of patients with stage 5 CKD who were admitted for planning their first dialysis as a treatment, which makes it different from the previous studies. We also objectively assessed the volume status of the patients at the time of echocardiography with BIS. Diastolic dysfunction is common among CKD patients, and worsens with increases in volume, culminating in both structural changes (such as cardiac stiffness) and functional crises (such as heart failure) [28, 29]. Biomarkers that detect volume overload at the early phase and allow timely intervention are necessary because fluid overload is a modifiable risk factor. Since OH/ECW measured by BIS is associated with echocardiographic parameters related to diastolic dysfunction, regular estimation and proper therapeutic approach for fluid overload may help reduce diastolic dysfunction in CKD patients. Further research may be needed to validate the consistency of this association across other stages of CKD.

## Supporting information

S1 Table Correlation of OH and OH/ECW with serum laboratory parameters. (DOCX)

## Abbreviations

CRP: C-reactive peptide
DBP: diastolic blood pressure
ECW: extracellular water
eGFR: estimated glomerular filtration rate
ICW: intracellular water
LA: left atrium
LAVI: left atrium volume index
LVEDD: left ventricular end-diastolic dimension
LVEDV: left ventricular end-diastolic volume
LVEF: left ventricular ejection fraction
LVFS: left ventricular fractional shortening
LVMI: left ventricular mass index
NT-proBNP: N-terminal pro-hormone of brain natriuretic peptide
OH: overhydration
SBP: systolic blood pressure
TBW: total body water
