# Supplementary material for: Association between OH/ECW and echocardiographic parameters in CKD5 patients not undergoing dialysis
Source: PLoS One. 2018 Apr 9;13(4):e0195202. doi: 10.1371/journal.pone.0195202 (PMC5891010; doi:10.1371/journal.pone.0195202)
Supplement: S1 Table — (DOCX) [file pone.0195202.s001.docx]

**S1 Table.** Correlation of OH and OH/ECW with serum laboratory parameters

| variables | OH | | OH/ECW | |
| --- | --- | --- | --- | --- |
|  | Corr. coeff. | P-value | Corr. coeff. | P-value |
| Age (years) | -0.19 | 0.03 | -0.12 | 0.17 |
| NT-proBNP (pg/mL) | 0.30 | 0.001 | 0.40 | <0.001 |
| Hemoglobin (g/dL) | -0.16 | 0.07 | -0.22 | 0.012 |
| CRP (mg/L) | 0.12 | 0.20 | 0.16 | 0.08 |
| Protein (g/dL) | -0.46 | <0.001 | -0.46 | <0.001 |
| Albumin (g/dL) | -0.51 | <0.001 | -0.54 | <0.001 |
| eGFR (mL/min/1.73 m^2^) | 0.12 | 0.19 | -0.04 | 0.66 |
| Ca (mg/dL) | -0.19 | 0.03 | -0.24 | 0.008 |
| P (mg/dL) | 0.10 | 0.25 | 0.16 | 0.07 |

Corr. coeff., correlation coefficient
